# Endogenous electromagnetic forces emissions during cell respiration as additional factor in cancer origin

**DOI:** 10.1186/s12935-016-0337-y

**Published:** 2016-07-28

**Authors:** Abraham A. Embi

**Affiliations:** 13442 SW 102 Lane, Miami, FL 33186 USA

## Abstract

**Background:**

Seven decades ago, a seminal paper by Dr. Denham Harman in (J Gerontol 11(3):298–300, 1956), introduced a theory stating that there are good reasons for assuming that endogenous irradiation in the living cells could lead to cancer via an obscure mechanism. The main purpose of this manuscript is to shed some light in said mechanism by proposing a five-step eukaryotic cell cancer triggering cycle. In other words, a new factor is introduced, namely the recently found emissions of electromagnetic forces (EMFs) as a possible causing agent in diseases, including cancer.

**Methods:**

Introduced is an eukaryotic cell cancer inducing cycle. It includes five sequential steps of endogenous biological process that are backed by published scientific reports.

**Results and Discussion:**

It is a known fact that in order to achieve homeostasis, toxic reactive oxygen species (ROS) i.e. H_2_O_2_ molecules are broken down by the protein enzyme catalase. During this reaction EMFs are generated (Embi in AIS Physics 2(3):226–230, 2016). The EMFs recording breakthrough was possible due to the introduction of a novel table top microscopy technique to detect EMFs by using Prussian Blue Stain and nano-sized iron particles. There are different roots in molecular and clinical biology through which DNA damage could be programmed, EMFs emitted (during cell respiration) are herein proposed as an additional cause.

## Background

The etiology and immortality of cancer cells has eluded the scientific community worldwide for times in memorial [1]. Ever since Nobel laureate Otto Warburg proposed his hypothesis, the current paradigm in cancer cells origin is that cancerous cells exhibit greater energy consumption than normal cells supplied by the fermentation of sugars i.e. glycolysis [2]. Basically his findings have lead generations of scientist to find a cancer cure by eliminating the cancer tissue energy sources.

Otto Warburg’s statement as to the prime cause of cancer is stated below: “Summarized in a few words, the prime cause of cancer is the replacement of the respiration of oxygen in normal body cells by a fermentation of sugar”. Warburg [3]. Moving a step forward, and using the Warburg Principle as a platform, a new “prime cause” is introduced as stated below: “One additional cause of the origin of cancer is directly related to exogenous and/or endogenous EMFs affecting the DNA within the eukaryotic cell”.

## Presentation of the hypothesis

**Fig. 1:**
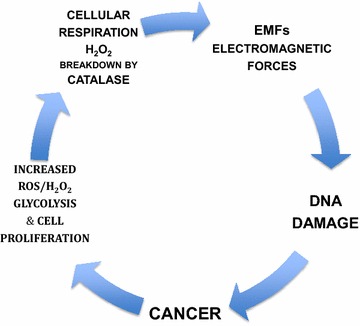
Proposed hypothesis leading to cancerogenesis by including the electromagnetic forces generated during cell respiration

Our aim in this manuscript is to move “Beyond Warburg” with a hypothesis introducing EMFs as a universal cellular cancer causing mechanism and consequent immortality. A five step cycle is depicted in (Fig. 1) which includes as main factors: (1) glycolysis emphasis on cell proliferation, (2) reactive oxygen species (ROS), (3) cellular respiration with emphasis on the breakdown of H_2_O_2_ by catalase, (4) EMFs emitted during cellular respiration (5) non-programmed DNA damage caused by the EMFs and (6) cancerogenesis. The hypothesis can be summarized as follows “One additional cause of the origin of cancer is directly related to exogenous and/or endogenous EMFs damaging the DNA within the eukaryotic cell”.

## Prior published work supporting the hypothesis

Characteristically cancer cells are known to exhibit a higher metabolic capacity supported by an increase in aerobic glycolysis that have enable others to theorize the linking of this high metabolic state with a prime causative cause for cancer. An example is attributing the upregulation of glycolysis to “microenvironmental acidosis requiring evolution to phenotypes resistant to acid-induced cell toxicity. Subsequent cell populations with upregulated glycolysis and acid resistance have a powerful growth advantage, which promotes unconstrained proliferation and invasion” [4]. There are also other published hypotheses regarding cancer genesis and cell proliferation that I find them to numerous to list in this manuscript. Now lets discuss the rationale behind the five-step cycle in cancer genesis and immortality hereby introduced. Please refer to Fig. 1.

*Step 1*: Cancer cells are known to increase the level of reactive oxygen species (ROS), which in turn have been found to further increase glycolysis [5] and have a demonstrated increase in energy metabolism (read glycolysis) correlated with the proliferation capacity of cancer cells [6]. As a note of interest and relevant to the hypothesis (see step 3) is a correlation found between increased metabolism and biomagnetism in the human hair follicle dermal papilla area [7].

*Step 2*: This step is an essential part of the hypothesis. Aerobic cellular respiration is ever present in eukaryotic cells, toxic byproducts such as H_2_O_2_ accumulation is neutralized by the ubiquitous protein enzyme catalase present in the cells peroxisomes [8].

*Step 3*: As aforementioned, during cellular respiration the physical interaction of catalase with ROS is part of the cell’s defense mechanism in eliminating toxic byproducts. The breakdown of the H_2_O_2_ molecule by catalase, as well as catalase proper have been reported to emit EMFs [9] that could be another causative agent in diseases.

*Step 4*: In this step, the emphasis is placed in the link between DNA damage and cancer. It has been reported that the responses of deoxyribonucleic acid (DNA) to EMFs show that due to the DNA structure, it possesses a wide range of interaction with EMFs [10]. The authors of that paper concluded that this property could account for “increases in cancer epidemiology since DNA damage has been linked to cancer” [11].

*Step 5*: Cancer genesis and beginning of proliferation and immortality…. Step 1 resume.

## Summary

### Endogenous EMFs and cancer

Reported is documentation supporting a hypothesis for a proposed cycle leading to cancer genesis. Cancer cells are known to possess a greater rate of glycolysis than normal cells; additionally there is also an increase in ROS level, which in turn increases further glycolysis and cell proliferation. During cell respiration, the breakdown of the H_2_O_2_ molecule by catalase, as well as catalase proper have been reported to emit EMFs. DNA is intrinsically fractal in nature; this property increases the probability of DNA strands to break when exposed to EMFs. The findings of internally emitted EMFs during cell respiration then causes strands breaks in the sensitive DNA structure, thus potentially leading to cancer. It should be noted that the other leading items in cancer include genetics, tumor biology and environmental factors.

## Implications of the hypothesis

Further research is warranted exploring beyond the starving of the cancerous cells as a sole or combined therapeutic mode. Controlling of our own intrinsic chronic EMFs bombardment could perhaps shed some light in the cancer war.

## Abbreviations

BMFs: electromagnetic forces emitted by biological entities
DNA: deoxyribonucleic acid
EMFs: electromagnetic forces
Glycolysis: the metabolic pathway that converts glucose
H_2_O_2_: hydrogen peroxide
ROS: reactive oxygen species

## References

[1] Harman D (1956). Aging: a theory based on free radical and radiation chemistry. J Gerontol.

[2] Pelicano H, Martin DS, Xu RH, Huang P (2006). Glycolysis inhibition for anticancer treatment. Oncogene.

[3] Warburg O (1956). On the origin of cancer cells. Science.

[4] Fais S, Venturi G, Gatenby B (2014). Microenvironmental acidosis in carcinogenesis and metastases: new strategies in prevention and therapy. Cancer Metastasis Rev.

[5] Gatenby RA, Gillies RJ (2004). Why do cancers have high aerobic glycolysis?. Nat Rev Cancer.

[6] DePreter G, Danhier P, Porporato PE, Payen VL, Jordan BF, Sonveaux P, Gallez B (2016). Direct evidence of the link between energetic metabolism and proliferation capacity of cancer cells in vitro. Adv Exp Med Biol.

[7] Embi AA, Scherlag BJ (2015). Human hair follicle biomagnetism: potential biochemical correlates. J Mol Biochem.

[8] Tripathi DN, Walker CL (2016). The peroxisome as a cell signaling organelle. Curr Opin Cell Biol.

[9] Embi AA (2016). Cellular respiration oxidation reduction reactions electromagnetic fields emissions as possible causative agent in diseases: a chronic bombardment theory. Phys J..

[10] Blank Martin (2011). Reba goodman DNA is a fractal antenna in electromagnetic fields. Int J Radiat Biol.

[11] Wang H, Zhang X, Teng L, Legerski RJ (2015). DNA damage checkpoint recovery and cancer development. Exp Cell Res.

